# Light-Powered Micro/Nanomotors

**DOI:** 10.3390/mi9020041

**Published:** 2018-01-23

**Authors:** Hongxu Chen, Qilong Zhao, Xuemin Du

**Affiliations:** Institute of Biomedical & Health Engineering, Shenzhen Institutes of Advanced Technology (SIAT), Chinese Academy of Sciences (CAS), Shenzhen 518055, China; hx.chen@siat.ac.cn (H.C.); ql.zhao@siat.ac.cn (Q.Z.)

**Keywords:** micro/nanomotors, light-powered, manufacture, motion manipulation

## Abstract

Designed micro/nanomotors are micro/nanoscale machines capable of autonomous motion in fluids, which have been emerging in recent decades owing to their great potential for biomedical and environmental applications. Among them, light-powered micro/nanomotors, in which motion is driven by light, exhibit various advantages in their precise motion manipulation and thereby a superior scope for application. This review summarizes recent advances in the design, manufacture and motion manipulation of different types of light-powered micro/nanomotors. Their structural features and motion performance are reviewed and compared. The challenges and opportunities of light-powered micro/nanomotors are also discussed. With rapidly increasing innovation, advanced, intelligent and multifunctional light-powered micro/nanomachines will certainly bring profound impacts and changes for human life in the future.

## 1. Introduction

The homeostasis of biological systems and locomotion of organisms in nature have long been an inspiring topic of research [1]. Inspired by natural microorganisms, considerable efforts have been devoted to achieving artificial self-propelled micro/nanomotors (MNMs) [2,3,4,5,6,7,8,9,10,11,12], which bring about different areas of influential applications, such as environmental remediation [13,14,15,16,17,18], target drug delivery [19,20,21,22,23,24], and cell manipulation and isolation [25,26,27,28,29,30,31].

MNMs are micro/nanoscale machines capable of converting different energies into mechanical energy that drives machinery movement. The energy sources can be chemical energy, derived from chemical reactions [32,33,34,35], or various sources of external stimuli (such as, light, magnetic, ultrasonic or electric field) [31,36,37,38,39,40,41,42,43,44,45,46,47,48,49]. Chemically-powered MNMs can act as reactants and/or catalysts to trigger in situ chemical reactions, subsequently generating chemical gradients or bubbles to autonomously propel themselves in a fluid. Typical ones are propelled by the decomposition of hydrogen peroxide (H_2_O_2_) [50,51,52,53,54,55,56,57,58,59,60]. The HCl, N_2_H_4_, I_2_ and other fuels have been also reported in succession for MNM propulsion [61,62]. The variety of fuels for propelling chemically-powered MNMs effectively increases their scope of applications, which has been reviewed by Samuel Sánchez et al. [63]. MNMs driven by external physical stimuli have been also widely investigated, of which several reviews have highlighted the advances of different systems, such as light-driven MNMs [64], ultrasound-driven MNMs [65], magnetic-driven MNMs (powered by a rotating magnetic field and an oscillating magnetic field), and electric-driven MNMs (in a direct-current electric field or an alternating-current field) [66]. In addition, recent advances regarding the motion manipulation of these externally-stimulated MNMs by different approaches have also been reviewed [67]. Since these externally stimulated MNMs with preset motion behaviors, long lifetimes, and excellent biocompatibility have shown great promise in various fields of technology, with the design and development of MNMs enabling quick responses to stimuli and precise motion manipulation, which are of great significance and attracting broad interest in research.

**Figure 1:**
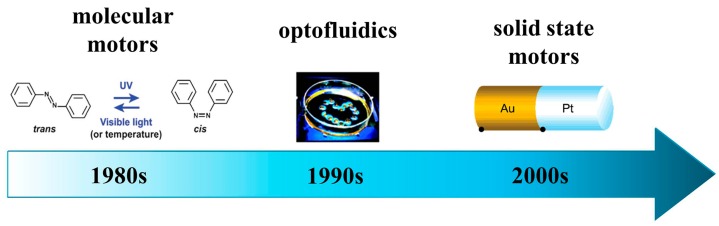
The milestones for the development process of light-powered MNMs: Molecular motors. Reproduced with permission [72]. Copyright 2012, Royal Society of Chemistry. Optofluidics. Reproduced with permission [73]. Copyright 2009, WILEY-VCH. Solid state motors. Reproduced with permission [74]. Copyright 2004, American Chemical Society.

Light is one of the most versatile power sources that is renewable and easy to control. With these unique features, light is an excellent candidate for the energy source to drive MNM movement, since the motion of elaborately-designed light-powered MNMs can be non-invasively controlled at highly precise spatial and temporal resolutions. Light-powered MNMs are propelled through converting light energy into mechanical energy, which is initiated from the development of molecular motors on the basis of light-responsive molecules. Significant milestones for the development of light-powered MNMs are summarized in Figure 1. Azobenzene-based artificial molecular machines were reported in the 1980s, propelled by the photoisomerization of chemical structures of azobenzene, were the first prototype of the initial light-powered MNMs. Inspired by kinesin, researchers have developed artificial walkers from DNA, and the molecules can take a step forward based on DNA cleavage and ligation. By using light as an energy source, DNA walkers can mimic the function of biological motors in cargo transport and biosynthesis [68,69,70,71]. Since then, various types of light-powered molecular motors were developed gradually. In the 1990s, researchers found that light can propel liquid droplet motion, which engendered a new research field named optofluidics, where the motion of a liquid is driven by optical forces, light-induced capillary forces or a combination of optical and electrical effects [72,73]. In 2004, an Au-Pt bimetal nanomotor reported by the Sen and Mallouk research group differentiated a new branch, namely solid state motors, which were inspired by the self-propelling plates reported by Whitesides et al. in 2002 [74,75]. The premise of the motion of solid state motors is the formation of an asymmetrical gradient field around the motors initiated by light. As the gradient field is unstable, a certain force around the motors is required to stabilize the gradient field to maintain its steady state in a fluid. In the end, this force drives the motors movement. Solid state light-powered MNMs offer the possibility to develop novel light-powered micro/nano robots with advanced properties and functions, making them an emerging topic in both the academic and industrial fields.

The objective of this review is to highlight various light-powered strategies to drive MNMs. In order to provide the reader with a general overview of the light-powered MNMs discussed in this review, we summarize some typical geometries, light sources, driving mechanisms and motion behaviors of light-driven MNMs, as shown in Table 1. By focusing on the fabrication of light-powered MNMs based on photoactive materials and structural design, we intend to discuss the importance of motion manipulation with regard to different light sources (e.g., ultraviolet (UV), visible and Near-Infrared (NIR) light) and motion behaviors. After briefly introducing the potential applications, we finally review the opportunities and challenges of the field.

## 2. Fabrication of Light-Powered MNMs

Light-powered MNMs are micro/nanodevices that can convert light energy into mechanical energy. The key to propelling the motion of light-powered MNMs is the formation of an asymmetrical gradient field around the motors initiated by light. To serve this purpose, light-powered MNMs are normally built either by employing photoactive materials (e.g., photothermal materials and photoisomerized materials) or by constructing asymmetrical structures/geometries (e.g., nanowires, Janus spheres, micro/nanotubes, microcapsules, etc.). In the following section, we will introduce the fabrication of light-powered MNMs based on photoactive materials and structural design, respectively.

### 2.1. Fabrication of Light-Powered MNMs Based on Photoactive Materials

**Figure 2:**
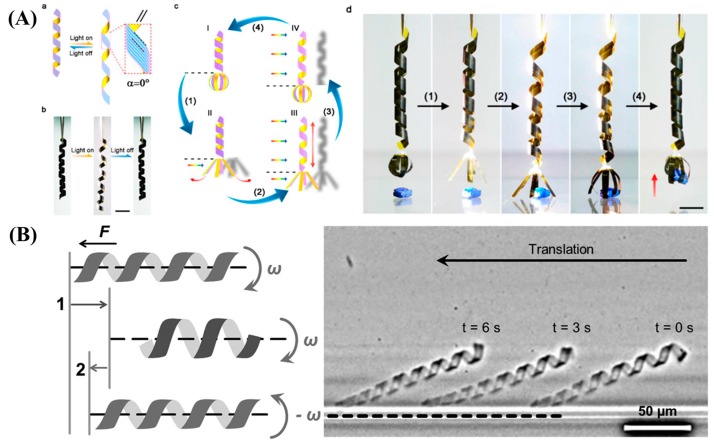
(**A**) Schematic illustration of helical Strip B (a). Photographs of helical Strip B before and after light irradiation (b). Schematic illustration of a mechanical arm completing a catching (releasing) movement (c). (d) Photographs of an object being lifted up by the mechanical arm. Scale bars, 1 cm in (b,d). Reproduced with permission [86]. Copyright 2016, American Chemical Society; (**B**) Illustration of the locomotion generated by non-reciprocal deformations of the helix (left); Directing the rotational motion to a linear translocation when the oscillating helix is confined close to a flat wall that impedes the rotation around the axis normal to the helix direction (right). Reproduced with permission [76]. Copyright 2016, WILEY-VCH.

Photoactive materials can absorb energy from the incident light and convert it into mechanical energy. Among them, photothermal materials, those that generate thermal effects under light irradiation, have been widely studied, by which many light-responsive actuators have been fabricated [85]. Furthermore, a series of tunable photo responsive actuators consisting of photothermal materials was demonstrated by Peng et al., which achieved an integration of complex movements triggered by light [86]. By adjusting the pre-programmed nanostructures, a light-manipulated mechanical arm was assembled and an energy harvesting system was used to execute complex but well-controlled motions. This mechanical arm was able to conduct movements of grasping/releasing and elongation/contraction manipulated by light illuminated areas. The four-step movements of the mechanical arm are shown in Figure 2A. The real-time response, remote controllability and light sensitivity of the mechanical arm offer high competency, as the arm can be adapted to perform different functions and be involved in different activities. Recently, the Martin Möller research group designed a new actuation mechanism for morphing a microswimmer with fast cyclic sequences of shape configurations, subsequently leading to translational motion, as shown in Figure 2B [76]. Light irradiation effectuated a thermal response for a purposefully designed hydrogel ribbon. Then the out-of-equilibrium response yielded precise and fast shape deformation with a rigorous and versatile control of complex motility modes, as needed for mobile microscale robots. They demonstrated the simple hydrogel ribbon motion in water. The ribbon not only followed a purposeful spatial configuration, but also underwent cyclic variations in its spatial configuration that followed a different forward and backward path in space and thus created a thrust to propel the hydrogel ribbon in water.

**Figure 3:**
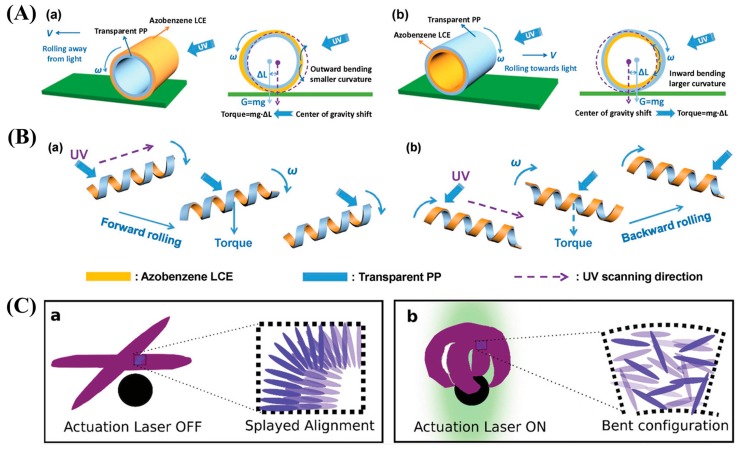
Light-controlled motion of liquid crystalline polymer. (**A**) Schematic showing (a) leftward and (b) rightward shift of the center of gravity in the wheel due to the UV-light-induced asymmetric deformation. Reproduced with permission [77]. Copyright 2017, WILEY-VCH; (**B**) Schematic of light-pushing forward rolling (a) and light-pulling backward rolling (b) of the helical ribbons due to UV-light-induced torque. Reproduced with permission [77]. Copyright 2017, WILEY-VCH; (**C**) Schematic of photonic microhand design (a) Illustration of a microhand and related mesogen alignment. (b) Illustration of the closed microfingers in response to an optical stimulus and the related change in molecular alignment. Reproduced with permission [87]. Copyright 2017, WILEY-VCH.

As an alternative photoactive material, liquid crystalline elastomer (LCE) has been attracting broad and growing interest in recent years because of their versatility in creating moving devices. Liquid-crystalline networks are smart materials that combine the anisotropic properties of liquid crystals with the good mechanical behavior of polymeric networks. They exhibit a shape change depending on the local alignment of the liquid-crystal director field inside the network by light illumination, inducing the mobility of LCEs. For example, Zhao et al. demonstrated the tunable photo-controlled motions of malleable azobenzene liquid crystalline polymer actuators [77], of which motion was driven by the UV light-triggered transformation of energy from stored mechanical strain energy in the polymer into mechanical force. This results in a variety of robust, tunable, and continuous motions at the macroscopic scale, as shown in Figure 3A,B. In another example, a photonic liquid-crystalline network microhand was reported by Wiersma et al., which was able to be remotely controlled by optical illumination, act autonomously and grab small particles resulting from their optical properties [87]. As shown in Figure 3C, the elastic reshaping properties of liquid-crystalline networks played a finger-like grasping action under light irradiation. Different deformations and motions could be also achieved by programming the alignment of liquid crystalline, which allow the polymer to perform a wider range of humanized actions in order to complete more delicate tasks.

**Figure 4:**
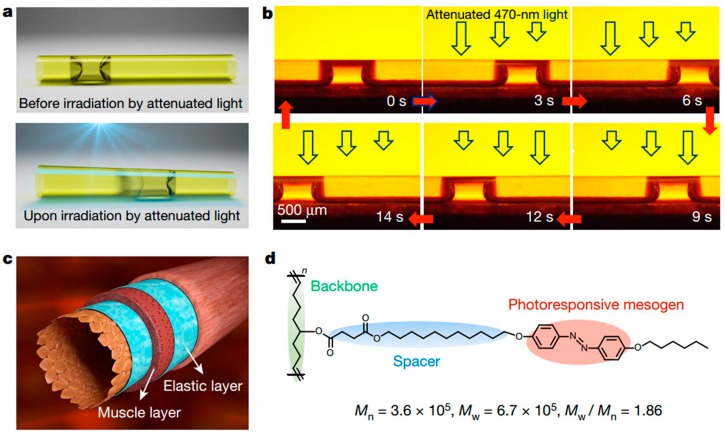
Design of tubular microactuators. (**a**) Schematics showing the motion of a slug of fully wetting liquid confined in a tubular microactuator (TMA) driven by photodeformation; (**b**) Lateral photographs of the light-induced motion of a silicone oil slug in a TMA fixed on a substrate; (**c**) Schematic illustration of the structure of artery walls; (**d**) Molecular structure of a novel linear liquid crystal polymer (LLCP). Reproduced with permission [78]. Copyright 2016, Nature Publishing Group.

Early studies of light-powered MNMs mainly focused on molecular machines based on molecular photoisomerization. Apart from the above-mentioned photo-controlled motions of liquid crystalline polymers, optofluidics enables more complex photo-powered motions. For example, Yu et al. reported a strategy to manipulate fluid slugs by photo-induced asymmetric deformation of tubular liquid crystal polymer microactuators, which induces capillary forces for liquid propulsion [78]. These microactuators are able to control a wide diversity of liquids over a long distance with controllable velocities and directions by light, as shown in Figure 4. The development of sophisticated light-powered MNMs by optofluidic approaches will be of great significance in the future.

### 2.2. Fabrication of Light-Powered MNMs with Different Geometries

**Figure 5:**
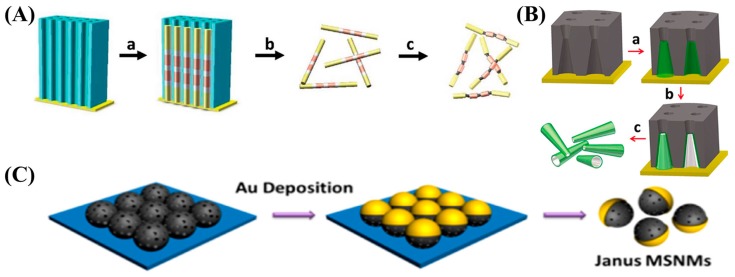
Fabrication schemes of template-assisted MNMs. (**A**) Schematic illustration of AAO template-assisted fabrication of the metal nanowires. Reproduced with permission [88]. Copyright 2011, American Chemical Society; (**B**) The fabrication process of PC template-assisted electrodeposition of micro/nanorockets. Reproduced with permission [58]. Copyright 2016, WILEY-VCH; (**C**) Fabrication scheme of spherical Janus MNMs. Reproduced with permission [81]. Copyright 2016, American Chemical Society.

Popular geometries of light-powered MNMs include nanowires, Janus spheres, micro/nanotubes, microcapsules, etc. The template method is a common method for fabricating light-powered MNMs with asymmetrical structures/geometries. Anodic alumina (AAO) membranes and polycarbonate (PC) containing cylindrical or conical pores have been used as preferred templates for the growth of nanowires or nanorockets by electrodeposition. The structure of nanowires or nanorockets could be controlled by the diameter of the membrane pores, deposition time and charges passed during its plating process. Different metals were used to form metallic nanowires or striped nanostructures with heterogenous composition and asymmetrical geometries by sequential deposition. Monodispersed metallic nanowires or nanorockets could be obtained by subsequently dissolving the membrane in the solvent (Figure 5A,B) [58,88]. Apart from nanowires, Janus spheres with distinct properties in the two faces of particles also favor the generation of gradient fields, thereby becoming interesting structures for fabricating light-powered MNMs. In order to obtain the half-coated particles, Janus spherical light-powered MNMs were fabricated by using monodisperse polystyrene (PS) or silica (SiO_2_) microspheres as the templates, followed by the deposition of metallic thin films on the microspheres (Figure 5C) [81]. To be specific, a suspension of PS or SiO_2_ microspheres was dropped onto a cleaned substrate to form a monolayer of the microspheres. The density of the monolayer covering the substrate could be controlled by varying the concentration of microsphere suspension, and the size of the microspheres could be adjusted as needed. Metal layers were then coated onto the monolayer of the microspheres by electron-beam evaporation or sputtering to obtain multi-metallic half-coated particles, which were finally released from the substrate to form Janus MNMs.

**Figure 6:**
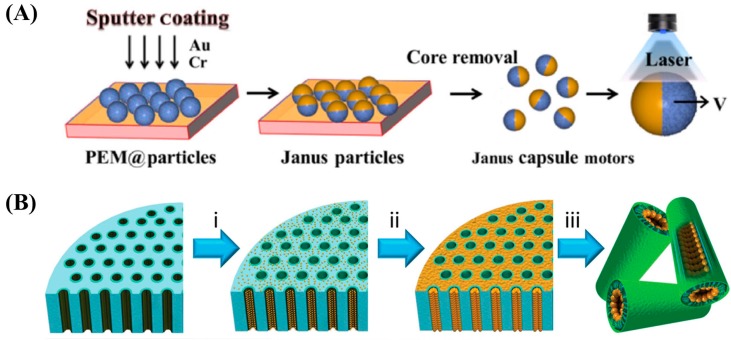
Schematic illustration of MNMs fabricated by the LbL method. (**A**) Scheme of light-triggered Janus capsule motors. Reproduced with permission [90]. Copyright 2014, American Chemical Society; (**B**) Illustration of the fabrication of tubular rockets: (i) LbL assembly of (PAH/PSS)_20_ films, and subsequent deposition of AuNPs into the pores of templates; (ii) Formation of AuNSs though surface-seeding growth method; (iii) Removal of the templates to release the rockets. Reproduced with permission [92]. Copyright 2015, Wiley-VCH.

The layer-by-layer (LbL) assembly technique, involving alternate deposition of positively and negatively charged polyelectrolytes, has been proven to be a versatile and convenient way to construct micro-/nanodevices with a precise structure and composition. He et al. have presented recent progress on the fabrication of MNMs [12]. By LbL assembly, polymers, nanoparticles, proteins and even anonymous assemblies can be conveniently integrated into or onto the LbL-assembled capsules or nanotubes through multiple weak interactions, including electrostatic interactions, hydrogen-bonds, coordination bonds, charge-transfer interactions, biologically specific interactions, and the combined interaction of the above forces, etc. He et al. fabricated the MNMs by LbL assembly of polyelectrolytes [89,90]. The negatively charged poly (styrenesulfonic acid) (PSS) and positively charged poly (allylamine hyhrochloride) (PAH) polyelectrolyte multilayers were adsorbed on the microspheres. Metal was subsequently deposited onto the (PSS/PAH)_5_-coated microspheres. The hollow Janus capsules partially covered by the Au layer could be obtained by removing the silica templates, as shown in Figure 6A [90]. Apart from Janus spheres, polymeric multilayer tubular rockets could be also prepared by the LbL method [91,92,93], as shown in Figure 6B. Briefly, the framework of the rockets was prepared by alternatively assembling PSS and PAH onto the inner walls of nanoporous polycarbonate membranes by LbL technique. Then negatively charged gold nanoparticles (AuNPs) were assembled into the (PSS/PAH)_20_-modified porous membranes via electrostatic interactions. The gold nanoshells (AuNSs) inside the rockets were formed through a seeding-growth procedure and finally the tubular rockets were released by dissolving the templates. The resulting rockets could perform NIR-triggered “on/off” motions in a remotely-controlled manner. However, the movement behavior of such a rocket is not stable. To solve this problem, He’s group fabricated a near-infrared-light-powered torpedo micromotor by the layer-by-layer sol-gel method, which performs stable movement in a straight line in various media [94]. In consideration of the manufacturing of light-powered MNMs, the LbL assembly method has the advantages of mass production and a convenient operation process. More ingeniously, some new methods such as colloidal lithography have recently emerged for fabricating light-powered MNMs with heterogeneous compositions and/or asymmetrical structures/geometries, which has proven to be a simple, inexpensive and versatile technique enabling rapid and large area patterning, as well as the formation of different conic materials with ordered structures [95,96]. The exploration of new manufacturing methods creates more possibilities for better control over the structures and geometries of light-powered MNMs, widening the functions of light-powered MNMs with more advanced properties.

## 3. Motion Manipulation of Light-Powered MNMs

For all types of MNMs, motion mode and motion manipulation are critical topics. Recently, many researchers reported the motion behavior of state-of-the-art light-powered MNMs together with their major propulsion mechanisms, including light-induced phoresis propulsion, bubble recoil, interfacial tension gradient, deformation propulsion, self-thermophoresis, and combination force [64,97,98,99]. For practical applications of light-powered MNMs, the stimulating light at a specific wavelength range (i.e., UV light, visible light and infrared light) is usually required to be in accordance with the nature of the MNMs. Hence, the effect of different light sources on the motion of light-powered MNMs is of high significance and will be reviewed in the following section. To achieve a higher level of motion manipulation for more sophisticated tasks, the controllability of the motion direction of the MNMs is another key aspect. Recent progress has demonstrated some novel light-powered MNMs enabling directional motion in remotely-controlled manners, which will also be summarized in this section.

### 3.1. Motion of MNMs Manipulated by Different Light Sources

#### 3.1.1. UV Light

**Figure 7:**
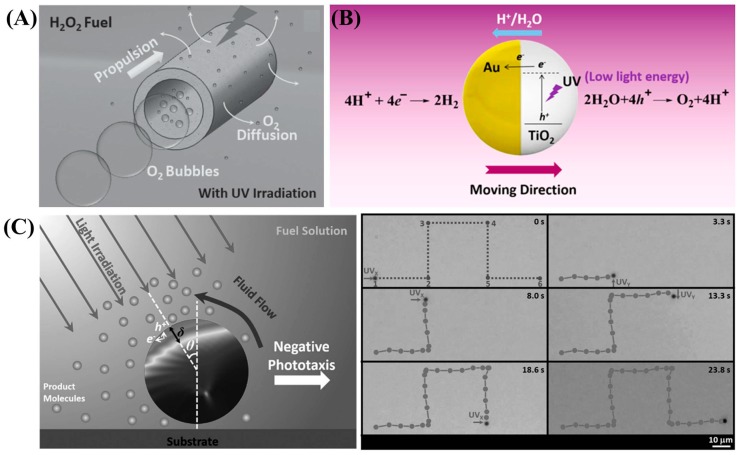
UV light-powered MNMs. (**A**) The UV-induced bubble propulsion mechanism of the TiO_2_ tubular microengine in H_2_O_2_ fuel, the generated O_2_ bubbles are ejected from a one-end large opening to propel the TiO_2_ tubular microengine. Reproduced with permission [100]. Copyright 2015, WILEY-VCH; (**B**) The mechanism schematic of TiO_2_-Au Janus micromotors powered by UV light in water. Reproduced with permission [79]. Copyright 2016, American Chemical Society; (**C**) The mechanism illustration of the phototaxis of a spherical TiO_2_ micromotor based on the limited penetration depth of light (graph on the left). Time-lapse images and the motion trajectory of a TiO_2_ micromotor in an aqueous solution containing 0.001 wt % H_2_O_2_ as fuel. The predesigned pathway for the micromotor is represented as dashed–dotted lines (graph on the right). Reproduced with permission [101]. Copyright 2017, WILEY-VCH.

For the first time, Guan et al. demonstrated a bubble-propelled photo-activated single component metal oxide tubular microengine by utilizing the photocatalytic H_2_O_2_ decomposition over TiO_2_ under UV irradiation [100]. Upon UV light irradiation, the photogenerated O_2_ molecules on the inner surface nucleate and grow into bubbles. Then the generated O_2_ bubbles are ejected from a one-end large opening to propel the TiO_2_ tubular microengine (Figure 7A). More importantly, the motion state and speed of the microengines can be reversibly, wirelessly, and remotely controlled by turning the “on/off” switch and regulating the intensity of the UV source. Figure 7B shows a highly efficient UV light-driven photocatalytic TiO_2_-Au Janus micromotor with wireless steering and velocity control. This Janus micromotor can be powered in pure water under an extremely low UV light intensity (2.5 × 10^−3^ W/cm^2^), and can reach a high speed of 25 body length/s at UV light intensity of 40 × 10^−3^ W/cm^2^ [79]. The propulsion of the TiO_2_-Au micromotors dominantly originates from the light-induced self-electrophoresis. Upon UV irradiation, charge separation occurs within the TiO_2_ and electrons are injected from the TiO_2_ conduction band into the Au hemisphere. Protons are produced from the oxidation of water at TiO_2_ and the resultant electrons are consumed during the reduction of protons at Au. The flux of H^+^ generates a fluid flow toward the Au hemisphere, generating a slip velocity and propelling the micromotors with the TiO_2_ hemisphere forward. In addition, Guan and Zhang et al. demonstrated a disruptive strategy to design micromotors by using isotropic structures. As shown in Figure 7C, the micromotors can continuously move, which induces a net concentration gradient of photocatalyzed products, independent of the random rotation of themselves. Both motion direction and speed were precisely controlled by UV irradiation. In this work, by taking advantage of the limited penetration depth of light in semiconductor materials, the asymmetrical surface chemical reactions on the isotropic semiconductor particles can take place, which induces concentration gradients of photocatalytic products to propel the micro/nanomotors. Due to their isotropic structures, the motion directionality of the as-developed micro/nanomotors is not interfered by their rotational Brownian diffusion or local flows, but always along the irradiated light direction [101].

#### 3.1.2. Visible Light

**Figure 8:**
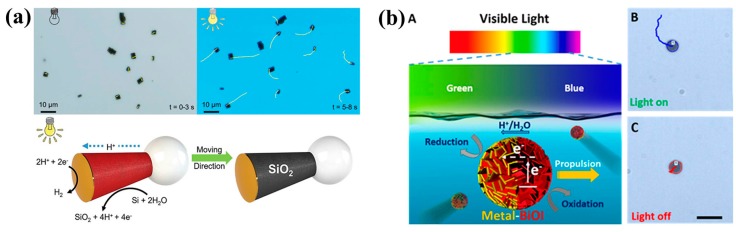
Visible-light-powered MNMs. (**a**) Trajectories of the Si-Au micromotors in water, from left to right, without illumination and with illumination at a light intensity of 13.6 mW mm^−2^ (top figures). Propulsion mechanism of the Si-Au micromotors activated by visible light in deionized (DI) water (down figure). Reproduced with permission [102]. Copyright 2017, Royal Society of Chemistry; (**b**) Mechanism illustration of visible-light-driven BiOI-metal Janus micromotors (A) and the movement trajectories of BiOI-metal Janus micromotors with and without light irradiation (B,C). Reproduced with permission [80]. Copyright 2017, American Chemical Society.

To the best of our knowledge, most of the existing light-powered MNMs are propelled autonomously by either UV or NIR light. In comparison with that, visible light may serve as an ideal external stimulus for propelling MNMs, as it is more easily available and convenient for operation. Recently, Li et al. reported visible-light-powered Si-Au micromotors, which could move in either deionized water or organic solvents without the addition of chemical fuels [102]. As shown in Figure 8a, the propulsion mechanism is the self-electrophoresis modulated by the photoconductivity of the amorphous silicon segment. Cai et al. presented visible-light-powered Janus micromotors based on BiOI microspheres with one hemisphere coated with a metal layer [80], propelled by the self-electrophoresis mechanism (Figure 8b). Although visible-light-powered MNMs possess many valuable properties for future biomedical and environmental applications, they still confront many challenges and further investigation may be required.

#### 3.1.3. NIR Light

**Figure 9:**
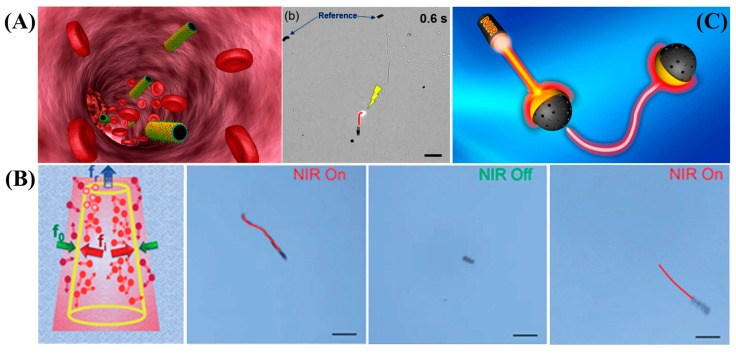
NIR light-driven MNMs. (**A**) NIR-induced launch of a microengine in 0.1% (*v*/*v*) H_2_O_2_ solution. Reproduced with permission [91]. Copyright 2014, American Chemical Society; (**B**) Schematic mechanism of NIR-driven rockets (Small arrows represent the inner and outer thermophoretic forces, and the large arrow indicates the direction of the resultant force) and time-lapse images of NIR controllable launch, stop, and restarted movement of the rocket. Reproduced with permission [92]. Copyright 2015, Wiley-VCH; (**C**) Schematic of NIR-driven Janus mesoporous silica nanoparticle motors. Reproduced with permission [81]. Copyright 2016, American Chemical Society.

In comparison with UV and visible lights, NIR light is of special interest in the consideration of biomedical applications since light absorption by biological tissues is minimal in this region and NIR light is safe for living organisms. He’s research group has focused on the construction of the NIR-driven MNMs, which pave the way to apply self-propelled synthetic MNMs in biomedical fields. In 2014, they demonstrated a new strategy for photothermally triggering the “on-demand” launch of gold-shell-functionalized polymer multilayer micromotors using a NIR laser at the critical concentration of peroxide fuel (Figure 9A). The process was based on the fact that the NIR illumination of the micromotors caused a spontaneous photothermal effect and thus a localized sharp increase in temperature around the micromotors. Accordingly, the increase in temperature induced the accelerated kinetics of the catalytic decomposition, the increased rates of mass transport, and the enhanced release frequency of oxygen bubbles [91]. Further, they presented a polymeric tubular rocket functionalized with AuNSs, which can move at a speed of up to 160 µm s^−1^ [92]. The strong plasma resonance absorption of AuNSs in the NIR region created localized temperature gradients on the inner and outer surfaces of asymmetric AuNSs (Figure 9B). The higher thermal gradient on the inner surface and the asymmetric structure of the rockets resulted in the difference in thermophoretic forces along the elongated axis of rockets, which in turn drove the rockets to move toward the direction of the front small-opening. Similarly, they constructed fuel-free, NIR-driven Janus mesoporous silica nanoparticle motors in 2016 [81]. As shown in Figure 9C, a localized photothermal effect on the Au half-shells resulted in the formation of thermal gradients across the Janus mesoporous silica nanoparticle motors. Thus, the generated self-thermophoresis could actively drive the nanomotors to move at an ultrafast speed upon exposure to an NIR laser. These NIR-powered MNMs demonstrate a novel strategy for overcoming the necessity of chemical fuels and exhibit significant improvement in the maneuverability of MNMs, which provide competent candidates for loading transportation in an eco-friendly manner.

#### 3.1.4. Multi-Wavelength Light

**Figure 10:**
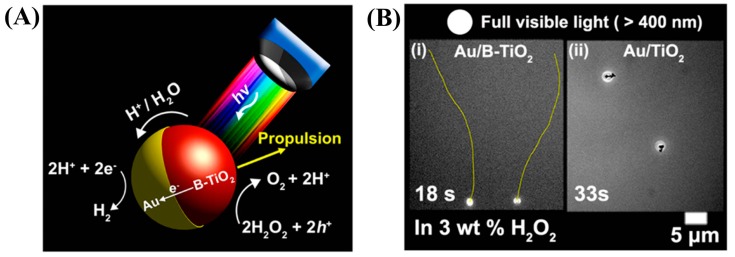
Full visible light (>400 nm) driven Au/B-TiO_2_ Janus micromotors. (**A**) Schematic of the propulsion mechanism of Au/B-TiO_2_ Janus micromotors; (**B**) Trajectories of (i) Au/B-TiO_2_ Janus micromotors over 18 s and (ii) Au/TiO_2_ Janus micromotors (control sample) over 33 s. Reproduced with permission [82]. Copyright 2017, American Chemical Society.

The above-mentioned light-powered MNMs are limited to the use of light at a specific wavelength. The narrow light absorption spectrum limits narrow ranges of wavelengths for locomotion. Multi-wavelength-light-powered MNMs were therefore gradually developed. For example, Nelson and Pane et al. reported multiwavelength light-responsive Au/B-TiO_2_ Janus micromotors [82]. As shown in Figure 10, the Janus micromotors showed directional motion under multiple light wavelengths including UV, blue, cyan, green, and red light not only in H_2_O_2_ solution but also in pure water. Because of their good photocatalytic activity at the entire spectrum of UV and visible light, their applications are considerably broad. In addition, Tang et al. have also successfully demonstrated a light-powered silicon nanowire-based nanomotor enabling response to multi-wavelength light (i.e., ultralow-intensity visible light and NIR light) [103]. The research into the multi-wavelength-light-powered MNMs is now just at the primary stage. With advances in materials and manufacturing technologies, novel smart light-powered MNMs, for which the motion behavior (e.g., motion speed, motion direction, etc.) can be manipulated and tuned by the light with different wavelengths, will be developed to perform complicated and multiple tasks intelligently in a controllable way.

### 3.2. Manipulation of Motion Behaviors

One important goal of MNMs is to manipulate their motion behaviors, mimicking those of live organisms. Organisms in nature can create highly complex collective behaviors through local interactions. The collective behaviors of flocking and schooling make organisms perform cooperative tasks. For example, motile bacteria exhibit organizational behaviors ranging from simple pairwise alignment and aggregation into swarms, to complex transport of other nonmotile species by symbiosis to detoxify their environment. Therefore, it is of great importance to control the collective motion behaviors of MNMs to biomimetic modes. Currently, most MNMs can only make curves or random movements unless an external magnetic field is applied [104,105]. The most challenging point is to control the movement speed and the movement direction of MNMs, which limits their applications. Compared to magnetic navigation, light navigation is an emerging method to manipulate MNMs.

**Figure 11:**
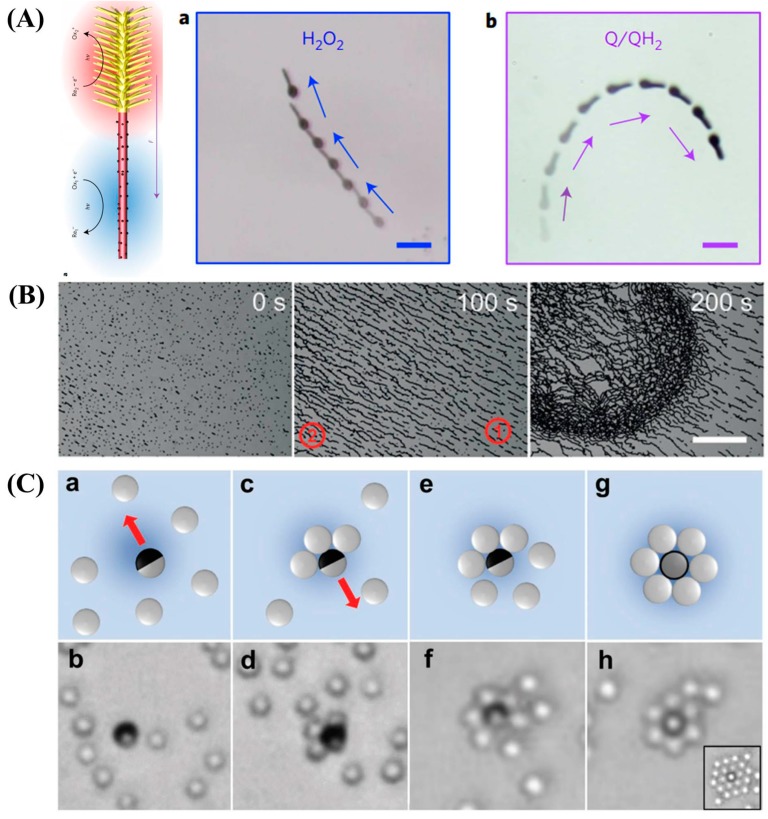
(**A**) Schematic of a Janus artificial microswimmer. Superimposed images of sequential frames show the migration of individual Janus nanotrees under global illumination in 0.1% H_2_O_2_ (a) and a mixture solution of 1,4-benzoquinone and hydroquinone (b). Reproduced with permission [83]. Copyright 2016, Nature Publishing Group; (**B**) Time-lapse optical images of collective behavior of peanut-shaped colloid motors under illumination of blue light. Reproduced with permission [84]. Copyright 2017, Wiley-VCH; (**C**) (a,b) Under UV illumination, an active particle adopts a tilted orientation, and moves with its TiO_2_ face leading. (c,d) Once trapped, passive particles preferentially attach to the TiO_2_ half (black region), and the active particle’s direction of propulsion reverses so that it moves toward its SiO_2_ face. (e–h) When more passive particles attach, the active particle usually reorients into a symmetric configuration with the active TiO_2_ surface facing up or down. Reproduced with permission [106]. Copyright 2017, WILEY-VCH.

Tang et al. presented a light-controlled programmable artificial phototactic microswimmer [83]. This microswimmer was Janus-nanotree-structured, containing a nanostructured photocathode and a photoanode at the opposite ends where cations and anions were released, respectively, subsequently propelling the microswimmer by self-electrophoresis. These microswimmers self-aligned at the direction of light propagation and mimicked the collective phototactic behavior of green algae in a solution (Figure 11A). By controlling the head and overall surface charges independently via chemical modification, the positive and negative phototaxis behaviors of microswimmers could be successfully programmed. Recently, the self-organization of a self-propelled peanut-shaped hematite colloid triggered by blue light was investigated by Qiang He [84]. Figure 11B shows the dynamic self-assembly of active colloid ribbons perpendicular to their long axis and the positive phototactic behavior of motile colloid ribbons in a solution of hydrogen peroxide fuel. The motion of colloid motors is ascribed to the diffusion-osmotic flow in a chemical gradient by the photocatalytic decomposition of hydrogen peroxide fuel. The phototactic behavior of colloid ribbons stems from the fact that the Gaussian beam distribution of light intensity leads to a higher rate of photocatalytic reaction in the center of the light spot and causes a larger hydrogen peroxide concentration gradient accordingly.

In addition to the aforementioned methods for achieving the programmed assemblies and collective motion manipulation of individual MNMs, a new method was developed and studied for the precise collective motion manipulation of light-powered MNMs [106]. It was found that self-propelled active colloids could induce the crystallization of passive silica colloids into well-controlled 2D assemblies when illuminated by UV light. The strength of the attractive interaction between the active colloids and the passive colloids, as well as the extent of the assembled clusters are modulated by the diffusiophoretic effects arising from a local chemical gradient activated by UV illumination, as shown in Figure 11C. Using this method, the collective motion of individual MNMs could be controlled, resulting in different assembly modes, e.g., isolated square, pentagonal, hexagonal, heptagonal clusters and some large assemblies with ordered and disordered translating symmetries, which offered a novel platform technology for making rationally designed colloidal clusters and crystals with controllable sizes, shapes, and symmetries.

## 4. Application Prospects

With their various excellent properties, light-powered MNMs have shown superior application prospects in environmental remediation and biomedicine. In terms of environmental remediation, light-powered MNMs have many advantages including remote operation, adjustable velocity and reutilization. For instance, specifically-designed light-powered MNMs modified by different active layers are able to adsorb certain metallic ions or remove specific oil pollutants, and are thereby promising for wastewater purification [13,14,16]. As for biomedical applications, soft infrared-powered MNMs are supposed to have significant potentials and merits. On the one hand, these soft infrared-powered MNMs can be fabricated by using polymers with proper mechanical properties and specific biological properties (e.g., biostability, biocompatibility, biodegradability and bioactivity), making them reliable and excellent candidates for implantation and clinical applications [31]. On the other hand, because of the deep penetration of infrared across live tissues, soft NIR-powered MNMs can be traced and triggered upon implantation in a noninvasive and remote way [21,64,89]. These advantages lay solid foundations for the preparation of advanced light-powered MNMs with novel properties and functions for diagnostic and therapeutic applications.

## 5. Conclusions and Outlook

In conclusion, research into light-powered MNMs has facilitated great progress in design and manufacture, showing different possibilities in various application fields. However, there are still many challenges for light-powered MNMs, including the limits of the operation environment, the adaptability of light wavelength, and the difficulty of motion direction control. Specifically, at present, the majority of light-powered MNMs are propelled in the fluids of H_2_O_2_ and H_2_O [80,82]. However, the toxicity of H_2_O_2_ severely restricts their scope of application and the speed of light-powered MNMs in pure water still needs to improve even though H_2_O is an ideal environment [80]. Besides, existing light-powered MNMs are mostly driven by UV and NIR irradiation [79,81], which may respectively cause damage to live organisms or unexpected thermal effects to the motor. The exploration of visible-light-powered MNMs, particularly enabling responses to visible light with different colors/wavelengths and desired driving efficiency, which remains a major challenge, is necessary but now relatively insufficient in the current investigations. Moreover, the precise control and manipulation of the directional motion of light-powered MNMs is a significant and challenging goal. The use of an external magnetic field is by far the most common and visible approach reported to realize the directional motion of MNMs to an intended direction or location. For light-powered MNMs, extensive efforts will still be made to achieve the precise regulation of their movement direction in innovative ways.

In the future, we believe that the exploration of highly efficient light response materials and the design of well-defined micro/nanostructures shall be of great importance, particularly for the development of advanced light-powered MNMs with excellent performance with low-cost, environmentally friendly and facile approaches. Furthermore, the research and development (R&D) of intelligent light-powered MNMs is an important direction via a combination of bio-inspired design and bio-inspired smart materials [64,107,108,109]. With intelligent abilities, these novel light-powered MNMs can perform complex tasks autonomously, and/or perform specific tasks in special environments, therefore hugely improving their scope of application.

## Figures and Tables

**Table 1:** Typical geometries, light sources, driving mechanisms and motion behaviors of light-powered MNMs.

	Geometries of MNMs	Light Source	Driving Mechanism	Motion Behavior	References
1	Hydrogel ribbon	Near-Infrared light	Photothermal effect	Translational motion	[76]
2	Wheel and spring-like ribbon	Ultraviolet light	Photoisomerization of azobenzene and strain energy	Controlled direction and speed	[77]
3	Tubular liquid crystal polymer	Blue light	Capillary forces arising from photodeformation	Controllable velocity and direction	[78]
4	TiO_2_-Au Janus micromotor	Ultraviolet light	Self-electrophoresis	25 body length/s	[79]
5	BiOI-metal Janus motor	Visible light	Self-electrophoresis	1.62 μm/s in pure water	[80]
6	Polymer multilayer rockets	Near-Infrared light	Thermophoretic force	High speed of 160 μm/s	[81]
7	Au/B-TiO_2_ Janus micromotor	Multiple light wavelengths	Self-electrophoresis	Maximus speed in H_2_O_2_: 30.1 μm/s	[82]
8	Nanotree	Ultraviolet light	Self-electrophoresis	Positive and negative phototaxis behaviors	[83]
9	Peanut-shaped colloid	Blue light	Diffusion-osmotic flow	Phototactic behavior	[84]
